# Canine Silica Urolithiasis in Mexico, Associated with the Concentration of Dissolved Silica in Tap Water

**DOI:** 10.1155/2021/6667927

**Published:** 2021-07-03

**Authors:** Claudia Iveth Mendoza-López, Javier Del-Angel-Caraza, María Alejandra Aké-Chiñas, Israel Alejandro Quijano-Hernández, Jody P. Lulich, María Vicenta Esteller-Alberich

**Affiliations:** ^1^Hospital Veterinario para Pequeñas Especies de la Facultad de Medicina Veterinaria y Zootecnia, Universidad Autónoma del Estado de México, Toluca, Mexico; ^2^Department of Veterinary Clinical Sciences, College of Veterinary Medicine, University of Minnesota, Saint Paul, Minnesota, USA; ^3^Instituto Interamericano de Tecnología y Ciencias del Agua-IITCA, Universidad Autónoma del Estado de México, Toluca, Mexico

## Abstract

Silica urolithiasis is infrequent in dogs, but in Mexico represents 12.9%. Our hypothesis is the consumption of high amounts of silicates in the diet, especially that dissolved in tap water. The objective of this study was to determine the concentrations of silica in the tap water in different geographical areas and their relationship with cases of silicate urolithiasis in dogs. From 179 cases of silicate urolithiasis, 98.9% were from dogs within a geographic area called the Trans-Mexican Volcanic Belt, which represents a cross shaft to the center of the country. Silica concentrations in tap water ranged between 3 and 76 mg/L, with a range of 27 to 76 mg/L, a mean of 49.9 ± 12 mg/L within the Trans-Mexican Volcanic Belt, and a concentration from 3 to 30 mg/L, with a mean of 16.4 ± 7 mg/L outside this area; these were significantly different (*p* < 0.001). These findings demonstrate that there is a geographic risk factor for silicate urolithiasis in urolith-forming dogs, related to the consumption of tap water with a high concentration of silica. Further studies are necessary to identify this same pathophysiological association in other species.

## 1. Introduction

Silica urolithiasis (SiU) is infrequent in dogs. In a global epidemiological study, SiU represented 0.69 to 0.74% of the samples analyzed in the reference laboratory [1]. Similar frequencies have been reported in local studies conducted in countries such as Brazil, United Kingdom, Spain, Portugal, and Canada [2–5]. In France, Hungary, and Ireland, it was not reported [6–8]. However, it was 6.7% in the United States [9], 8% in Switzerland [10], and 9.2–13.3% in Mexico [11–13]; the frequencies have been considerably higher.

It has been suggested that SiU is related to the urinary supersaturation of silicates (SiO_4_)^4−^ (SiO), derived from the consumption and intestinal absorption of different silicates present in the diet. It is common in cattle and sheep from the Great Plains of North America and in some areas of Australia, where it is related to the feeding of these species, namely, the consumption of forage grasses with a high SiO content [14]. In dogs, it has been associated with the consumption of low-quality pet food in which vegetable ingredients, e.g., corn gluten, rice husks, wheat husk, beet pulp, and barley, are added as a source of protein or soluble fiber that may have high SiO content [15]. In a report of native dogs in Kenya, this pathology was hypothesized in association with the consumption of corn and water with a concentration of silica (Si) of 20–30 mg/dl [16].

In humans, SiU clinical cases related to the chronic use of antacids containing magnesium trisilicate have been reported, so this is considered of medicinal origin [17]. However, a clinical case of a 10-month-old baby with SiU, without drug use, was associated with the consumption of water with a high concentration of Si [18]. Recently, it has been related to a possible geographical-cultural risk factor, as reported in West Africa, where SiU is associated with pica. Pica is a widespread practice in several population groups that consume clay as a condiment, which acts as a source of high SiO content in the diet [19].

In Mexico, SiU has been reported in populations of dogs that live in cities located within the geographical area called the Trans-Mexican Volcanic Belt (TMVB) [11–13], the largest Neogene volcanic arc in North America that encompasses 160, 000 km^2^ with a length of almost 1, 000 km in central Mexico. It is characterized as being a large mass of volcanic rocks composed of a high SiO content [20]. Although specific medications and diets have been associated with SiU in humans, we have not observed these risk factors in dogs forming SiU. We hypothesize that the drinking tap water from the aquifers in the TMVB in Mexico contains a high concentration of Si and is a geographic risk factor for the formation of uroliths in dogs [11–13].

The objective of this study was to determine the concentration of Si in the tap water in different regions of Mexico (populations located within and outside of the TMVB) and its relationship to SiU in the TMVB in dogs.

## 2. Materials and Methods

During the period from January 2016 to July 2018, we measured the concentration of Si in drinking water samples taken directly from the tap in different states of Mexico. We also analyzed the urolith database from the period 2005 to 2018 of the Urolith Analysis Laboratory of the Veterinary Hospital for Small Species of the Faculty of Veterinary Medicine and Zootechnics of the Autonomous University of the State of Mexico (UAL-HVPE-UAEM). Urolith samples were referred by veterinarians from different states of the country for analysis, accompanied by a registration form containing general and clinical information and the geographic location of clinical cases in the country. We count the registry data of the states where the SiU cases occurred; once located, the origin of the SiU cases and the water samples were grouped into two geographical areas: the central region, within the TMVB (ITMVB), and the region outside the TMVB (OTMVB), which, in turn, was divided into two areas (north and south).

### 2.1. Water Sample Collection and Preservation

The water samples were collected by opportunity sampling from the 32 states of Mexico; samples were taken directly from the tap in a 250 mL plastic bottle and preserved by refrigeration at 4°C until analysis [21].

### 2.2. Analysis of Si in Water

The concentration of Si in the water was determined by the method of colorimetry, with a system of reagents using the silicomolybdate method (Code 3687-SC; SMART2 colorimeter, LaMotte Company, MD, USA), and details on sample preparation methods were described in [21]. Briefly, in a glass tube containing the sample to be analyzed (10 ml of water), hydrochloric acid was added and mixed. Subsequently, ammonium molybdate and potassium hydroxide were added. The tube was then covered, mixed, and left to stand for 5 minutes. At the end of the 5-minute period, oxalic acid was added, and then, the tube was covered, mixed, and placed inside the colorimeter to scan the sample. Si forms a complex with ammonium molybdate in an acidic solution and produces a yellow color proportional to the amount of Si present. The Si concentration measurements were expressed in mg/L [21].

### 2.3. Statistical Analysis

The obtained data were stored in a database using the Excel program and analyzed with GraphPad Prism 6.0 (https://www.graphpad.com CA, USA). To determine the risk factors according to the geographic location of the stone-forming dogs, the control group was selected from the database of urolithiasis cases analyzed at the UAL-HVPE-UAEM; this included cases of stone-forming dogs of other types of uroliths, in the same period of time as the cases with a diagnosis of SiU; the *Xi*^2^, OR, and 95% confidence interval were determined. The mean, range, and standard deviation for the values of the concentrations of Si in the water were calculated. Differences in the concentration means between the geographical regions were determined using an ANOVA. Differences were considered significant when *p* < 0.05.

## 3. Results

A total of 1,383 uroliths from dogs were analyzed. Prevalence of each type of uroliths were struvite *n* = 611 (44.2%); calcium oxalate *n* = 372 (26.9%); silica *n* = 179 (12.9%); purines *n* = 57 (4.1%); cystine *n* = 17 (1.2%); calcium phosphate *n* = 13 (0.9%); and mixtures of minerals in mixed uroliths *n* = 105 (7.6%) and compounds *n* = 29 (2.1%). Therefore, silicate uroliths represented 12.9% (*n* = 179) and OU 87.1% (*n* = 1,204).

On the basis of the region of origin of patients who formed uroliths, 1184 (85.6%) cases were ITMVB, whereas 199 (14.4%) cases were OTMVB. Of the 179 cases of SiU, 177 (98.9%) were found in the states located ITMVB (Table 1); only two cases (1%) were in two states in the north region of the country located OTMVB (Table 2). Dogs from ITMVB had a significantly higher risk (OR = 17.31 95% CI = 4.261–70.35; *p* < 0.05) of forming silica uroliths.

**Figure 1:**
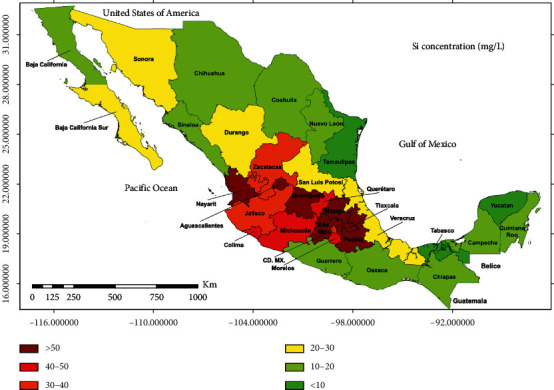
Mexican states with cases of SiU and regions with higher concentrations of Si in tap water.

A total of 168 drinking tap water samples were analyzed, taken directly from the tap, corresponding to 32 states in Mexico. Si concentrations in drinking water ranged between 3 and 76 mg/L (Figure 1).  Table 3 shows the number of samples analyzed for each state, as well as the mean concentration of Si in the tap water.

**Figure 2:**
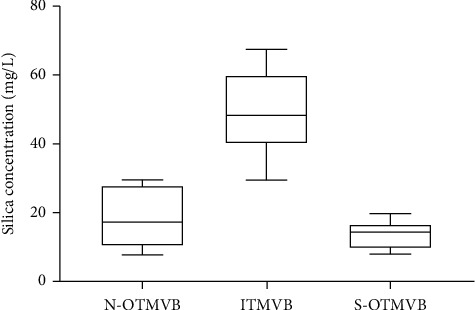
Mean values and standard deviation of the concentration of Si in the tap water of different geographical areas. N-OTMVB: north region outside of the Trans-Mexican Volcanic Belt; ITMVB: within the Trans-Mexican Volcanic Belt; and S-OTMVB: south region outside of the Trans-Mexican Volcanic Belt.

The highest concentrations of Si in the water occurred in the ITMVB region (Figure 2), with a range of 27 to 76 mg/L and a mean of 49.9 ± 12 mg/L. For the case of the OTMVB region, the Si concentration ranged from 3 to 30 mg/L, with a mean of 16.4 ± 7 mg/L, and there were statistically significant differences between the regions (*p* < 0.0001).

In particular, for the two OTMVB areas, the concentration of Si in the water ranged from 3 to 30 mg/l, with a mean of 18 ± 8.2 mg/L in the north region, and ranged from 6 to 22 mg/L, with a mean of 13.9 ± 4 mg/L in the south region. There were no significant differences between these two geographic regions OTMVB (*p*=0.64).

## 4. Discussion

The high frequency of SiU in dogs in Mexico, as well as in other animal and human populations in other parts of the world, might be associated with a geographic risk factor [13, 19]. Given the 179 cases of SiU in Mexico, we were able to identify its geographical distribution [13]. We noted that 99% of cases were in ITMVB. This area is a chain of volcanoes that extends transversely from coast to coast, from the Revillagigedo Islands in the Pacific Ocean to the Gulf of Mexico [22]. In urolith-forming dogs, living in this geographical area was a significant risk factor for forming SiU.

Urinary supersaturation with this mineral is essential for the formation of uroliths; the excreted amount of SiO in the urine is proportional to the amount ingested in the diet from solid food and water [23]. Once ingested, Si is absorbed and reaches the blood plasma, passes to the tissues, and is excreted by the kidney through glomerular filtration, which is not reabsorbed by the tubules, and eliminated in the feces [24]. The urinary excretion of Si is in the form of orthosilicate, to be later transformed into silicon dioxide (SiO_2_) when it comes into contact with acidic urine [25].

In solid food, SiO is found as colloid molecules of the type “colloidal silica.” This is a polymeric species, with large, aggregated, and charged molecules, which have a low absorption in the gastrointestinal tract because the particles must be decomposed into a soluble monomer so that they can be absorbed; the rate of hydrolysis in the gastrointestinal tract is slow compared to the window of opportunity for absorption in the small intestine [26]. In certain grain and cereal products, there is a silicon fitolitic type, which is digested and is easily absorbed in the gastrointestinal tract, and several vegetables with less soluble SiO are used in pet foods such as corn gluten, wheat, or rice husk; this can interact with the concentrations of Si in the blood. In our study, it was not possible to identify an association between SiU and the type of food consumed by the dogs. Information about the diet consumed by the dogs was incomplete.

In water, Si particles are predominantly of monomeric species, such as orthosilicic acid (H_4_SiO_4_) [27]; they are small molecules with a highly soluble neutral charge, presenting high gastrointestinal absorption, and are expelled rapidly in the urine [28]. The higher Si concentration in the tap water in the TMVB in Mexico is consistent with the concentrations of Si in water that are linked to the geological characteristics of the area. Water with a high concentration of Si has been found in the aquifers in volcanic areas. In this study, the majority of the dogs consumed tap water. Only one dog was provided bottled water exclusively, and another consumed both tap water and bottled water; both dogs lived in ITMVB. Although the dogs that consumed another source of water also developed SiU, which may be related to the bottled water, they probably consumed a high concentration of Si. One study analyzed bottled water consumption and observed that those containing high concentrations of Si came from volcanic zones [29]; therefore, it is possible that bottled water consumed by these dogs was packaged within the same region.

With these findings, we observed that stone-forming dogs living within ITMVB have a higher risk of developing SiU because the drinking water of this region contains a higher concentration of Si (Figure 1). In humans, the frequency of SiU is unknown because conventional techniques, such as the metabolic profile of lithiasis performed in humans, do not allow the identification of this mineral [30] and for the identification of SiO in uroliths, it is necessary to use analysis techniques, such as infrared spectroscopy. The cases of SiU in the dogs studied herein can be a sentinel epidemiological model for the presentation of urolithiasis in humans in Mexico.

## 5. Conclusions

From these epidemiological data, we conclude that dogs living in the Trans-Mexican Volcanic Belt of Mexico have a higher risk of forming silica uroliths because of the high concentrations of Si in the water in this area. Other sources of silica such as local vegetation, dirt, and air are also potential sources of silica, but were not tested in our study.

## 6. Limitations

For the study period, it was not possible to obtain exclusive water samples from the patient with urolithiasis and data such as volume or frequency of ingestion of tap water and factors that together with the high concentrations of silica in tap drinking water can influence the development of SiU.

## Figures and Tables

**Table 1:** Proportion of SiU in states within and outside the Trans-Mexican Volcanic Belt.

States ITMVB	*n* SiU/OU	%	States OTMVB	*n* SiU/OU	%
Aguascalientes	6/9	66.6	Chihuahua	1/40	2.5
Colima	7/58	12.0	Nuevo Leon	1/54	1.8
Guanajuato	10/37	27.0	Other states	0/103	0
Hidalgo	7/9	77.7			
Jalisco	18/177	10.1			
Mexico City	58/463	12.5			
Mexico state	53/139	38.1			
Michoacan	7/47	14.9			
Morelos	4/6	66.6			
Nayarit	1/7	14.2			
Puebla	3/28	10.7			
Queretaro	3/10	30			
Other states	0/17	0			

ITMVB: within the Trans-Mexican Volcanic Belt; OTMVB: outside of the Trans-Mexican Volcanic Belt; SiU: silica urolithiasis cases; OU: other urolithiasis cases.

**Table 2:** Distribution of silica urolithiasis cases in different geographical areas of Mexico.

	SiU *n* (%)	OU *n* (%)	Total *n* (%)
ITMVB	177 (15)	1007 (85)	1184 (100)
OTMVB	2 (1)	197 (99)	199 (100)

Totals	179	1204	1383

ITMVB: within the Trans-Mexican Volcanic Belt; OTMVB: outside of the Trans-Mexican Volcanic Belt. SiU: silica urolithiasis; OU: other urolithiasis.

**Table 3:** Number of tap water samples analyzed by Mexican states, including the mean values, standard deviations, and range of Si concentration according to the state.

State ITMVB	Si concentration (mg/L)				State OTMVB	Si concentration (mg/L)			
	n	Mean	SD	Range		n	Mean	SD	Range
Aguascalientes	4	67.5	6.9	59–76	Baja California S	3	28.7	0.5	28–29
Mexico City	5	38.6	2.4	36–42	Baja California N	4	12	9.3	7–26
Colima	3	42	13.4	27–53	Campeche	4	16	1.8	14–18
Mexico state	17	58.5	1.8	42–76	Chiapas	4	13.2	4.0	10–19
Guanajuato	5	53.4	6.1	47–63	Chihuahua	9	18.4	4.3	11–27
Hidalgo	6	65.5	3.3	62–73	Coahuila	3	10.6	3.0	8–14
Jalisco	8	36.7	0.5	34–40	Durango	2	29.5	0.7	29–30
Morelos	3	41.6	4.0	38–46	Guerrero	6	14.4	5.2	6–20
Michoacan	5	48.2	5.8	43–55	Nuevo Leon	5	9.4	2.19	8–13
Nayarit	5	67	9.8	52–76	Oaxaca	4	19.7	4.5	13–22
Puebla	7	59.8	10.0	34–73	Quintana Roo	6	16.7	7.0	6–22
Queretaro	6	45.5	9.1	37–61	San Luis Potosi	4	20.5	2.1	10–29
Tlaxcala	3	55	2	53–57	Sinaloa	5	16.2	1.7	14–19
Veracruz	8	29.4	2.5	27–34	Sonora	5	27.6	1.6	25–29
Zacatecas	3	40	1	39–41	Tabasco	5	8	2	6–10
					Tamaulipas	4	7.7	3.3	3–10
					Yucatán	7	9.6	3.2	7–15

ITMVB: within the Trans-Mexican Volcanic Belt; OTMVB: outside of the Trans-Mexican Volcanic Belt.

## Data Availability

The data used to support the findings of this study are available from the corresponding author upon request via email, dlangel@uaemex.mx.
